# Optimizing tissue donation in critical care and emergency department at a university hospital

**DOI:** 10.1186/2197-425X-3-S1-A907

**Published:** 2015-10-01

**Authors:** D Daga Ruiz, F Segura González, J Pérez Vacas, A Fernández Porcel, A Puerto Morlan

**Affiliations:** Hospital Virgen de la Victoria, Coordinación de Trasplantes, Málaga, Spain

## Introduction

The increase of human tissue clinical use has lately generated a relevant donors demand, which in terms of tissue donation is not limited to brain dead donors, but generalized to cardiac arrest donors (multi-tissue donor) and alive donors.

Donated organs and tissues both come from an alive or dead donor, which implies similarities in terms of ethical requirements. However, there are several aspects that differentiate both protocols and make a difference in the approach.

- No time urgency.
- Storage.
- Tissue extraction can be adjusted to clinical demands and tissue bank availabilities.

These distinguishing factors previously mentioned make tissue donation after cardiac arrest possible.

## Objectives

The tissue donor after death has as origin a patient who dies from cardiac arrest. In this scenario, tissue donation inclusion in end life care pathway is essential in order to enhance the tissue donor pool after death.

## Methods

During the period 2004-2014, an awareness raising initiative in tissue donation process and the multi-tissue donor was carried out, that consisted on:

Daily routine visit to Observation and Intensive Care departments by the transplant coordinator and/or intensivist on call, in order to evaluate patients who had been admitted in these areas, their pathology and potential inauspicious prognosis.

Mortality review within the past 24 hours

Ominous prognosis admissions review within the past 24 hours

Training programmes and awareness raising initiatives in donation process for hospital health professionals, focusing on Emergency Medicine and Critical Care departments. Solid organ and tissue donation training programmes, Reference Guide to consent for donation, Hospital sessions, donation and transplant activity registers sessions per department each 3 months.

## Results

During the previously mentioned period of time in which this new proactive attitude towards donation has been carried out, donation activity has constantly increased, going from no multi-tissue donors before 2004 and 2 in 2006 to 26 in 2014; in fact, 6% of the total number of deaths occurred in 2014 in the emergency department and intensive care unit led to multi-tissue donation (2 or more tissues donated per donor), and health professionals established the exitus -donation link.

## Conclusions

In the last years we have witnessed a spectacular progress in the clinical use of human tissue in different areas which has generated a considerate tissue donor demand. Tissue donors generated by patients who die of cardiac arrest and are not candidates for solid organ donation (the majority of them) suppose a relevant source of tissue donors that should always be considered.

## Grant Acknowledgment

We would like to thank donor relatives for their priceless contribution and understanding. Thanks to them thousands of people are born again.
